# OptPipe - a pipeline for optimizing metabolic engineering targets

**DOI:** 10.1186/s12918-017-0515-0

**Published:** 2017-12-21

**Authors:** András Hartmann, Ana Vila-Santa, Nicolai Kallscheuer, Michael Vogt, Alice Julien-Laferrière, Marie-France Sagot, Jan Marienhagen, Susana Vinga

**Affiliations:** 1 IDMEC, Instituto Superior Técnico, Universidade de Lisboa, Av. Rovisco Pais 1, Lisbon, 1049-001 Portugal; 2 Institute of Bio- and Geosciences, IBG-1: Biotechnology Forschungszentrum Jülich GmbH, Jülich, D-52425 Germany; 3 EPI ERABLE, Inria Grenoble, Rhône-Alpes, France; 4 Université de Lyon, F-69000, Lyon; Université Lyon 1; CNRS, UMR5558, Laboratoire de Biométrie et Biologie Evolutive, Villeurbanne, F-69622 France

**Keywords:** Metabolic engineering, Metabolic networks, Optimization, Software, Rank product

## Abstract

**Background:**

We propose OptPipe - a Pipeline for Optimizing Metabolic Engineering Targets, based on a consensus approach. The method generates consensus hypotheses for metabolic engineering applications by combining several optimization solutions obtained from distinct algorithms. The solutions are ranked according to several objectives, such as biomass and target production, by using the rank product tests corrected for multiple comparisons.

**Results:**

OptPipe was applied in a genome-scale model of *Corynebacterium glutamicum* for maximizing malonyl-CoA, which is a valuable precursor for many phenolic compounds. In vivo experimental validation confirmed increased malonyl-CoA level in case of *ΔsdhCAB* deletion, as predicted *in silico*.

**Conclusions:**

A method was developed to combine the optimization solutions provided by common knockout prediction procedures and rank the suggested mutants according to the expected growth rate, production and a new adaptability measure. The implementation of the pipeline along with the complete documentation is freely available at https://github.com/AndrasHartmann/OptPipe.

**Electronic supplementary material:**

The online version of this article (doi:10.1186/s12918-017-0515-0) contains supplementary material, which is available to authorized users.

## Background

In the context of microbial cell factories, the optimization of genetic backgrounds has been carried out through classical mutagenesis followed by selection techniques and rational metabolic engineering strategies. The goal of metabolic engineering is to introduce a set of cooperative genetic modifications that rewire the microbial metabolism towards the production of a target compound. This is accomplished by elimination, addition or modification of metabolic reactions, resulting in a strain that shows increased target compound production, which, in an ideal case, is coupled with growth. Although promising, rational-guided metabolic engineering strategies can have unpredicted effects in distant parts of the metabolism and rely on biological intuition. For these reasons, the field has accommodated the use of genome-scale stoichiometric models to guide metabolic engineering strategies [1–3]. A large emphasis has been put into the development of optimization methods that can predict beneficial genetic modifications for the increased production of a given compound of interest (target), mostly based on constraint-based modeling, where the aim is to couple chemical production to growth [1, 4, 5]. In this setting, Flux Balance Analysis (FBA) is the most widely used formulation, where the steady-state linear programming problem is solved by maximizing a cellular objective function which is most commonly the growth rate [6]. Other frameworks have been established to study the metabolism at a higher level, taking into account the enzyme kinetic parameters of the system (Metabolic Control Analysis, MCA) and/or transcription and signaling pathways (Hierarchical Control Analysis, HCA) [7, 8]. However, the large volume of information required for the construction of such models prevents its utilization at the genome-scale for the prediction of metabolic engineering strategies.

This work is focused on the problem of predicting relevant knockout strategies to increase target production when a genome-scale stoichiometric model is available. One of the first tools devised to meet this problem was OptKnock, which is formulated as a bi-level problem that can be translated into a single-level mixed integer problem and that delivers a list of recommended deletion strategies to increase chemical production [9, 10]. OptReg, an extension of OptKnock that allows the prediction of also down- and upregulation strategies, followed [11]. Subsequently, RobustKnock was developed to meet the problem of the overly optimistic predictions delivered by the latter frameworks. This was avoided by formulating a tri-level problem where the worst-case (minimal) target production is maximized given that the biomass is maximized [12]. The above-mentioned algorithms are based on FBA where the cellular objective function is maximization of growth. However, as it is not always the most accurate formulation to calculate the flux distribution upon genetic manipulation, the MOMA framework can be useful to deal with this problem. It predicts the flux distribution of the genetically modified organism by relying on the assumption that its fluxes undergo a minimal redistribution when compared to the wild type [13]. OptGene is a different strategy that relies on evolutionary programming to find knockout strategies that increase the objective function BPCY (Biomass Product Coupled Yield), a surrogate for productivity that takes into account the chemical production and the growth rate [14]. This tool is also implemented in the open-access workbench OptFlux [15]. An alternative approach to the same problem that combines information from flux variability profiling and metabolite centrality is RobOKoD, where knockout, overexpression and dampening strategies are suggested based on the profiling of every reaction in the model and its relation to the target production [16].

Each of these methods is based on different approaches and rationales, therefore leading to distinct solutions. In fact, one key issue is the multitude of obtained results, known to be dependent on the specific algorithms and the software implementation used. In order to address this problem, a consensus-based approach is here proposed and developed. The method is based on running several optimization procedures for knockout prediction and analyzing *a posteriori* the consensus solutions obtained. From the available optimization methodologies, OptKnock was chosen, as it is one of the most used methods, as was the corresponding pessimistic prediction strategy, RobustKnock. In addition, OptGene and RobOKoD were also introduced in this procedure, as they represent very different approaches. Naturally, each method has its advantages and disadvantages: OptKnock and RobustKnock are optimization-based methods, and return an optimal solution to the defined mixed integer linear problem (MILP). Notice that there might be numerous solutions with optimal objective value, out of which only one is returned. In contrast, there are heuristic methods, like the Evolutionary Algorithms and Simulated Annealing implemented in the software tool OptFlux [15] and OptGene [14], respectively. These methods allow nonlinear/non-convex objective functions, and return a set of candidate mutants. Heuristic methods do not guarantee that any of the returned solution is globally optimal, but identify the best performing solutions from the populations where the objective function was evaluated. RobOKoD also returns a list of scored solutions based on a three-step method. See the detailed formal description of the different methods in the Additional files 1 and 2.

The rationale of using multiple optimization methods is to have rankings of hypotheses that may provide confidence in particular sets of proposed genetic alterations from various aspects. Using this strategy, it is possible to combine several criteria, taking into account possible performance indexes simultaneously; for example, the maximal predicted target compound production, the minimal predicted target compound production and the distance from the wild-type flux distribution. In this sense, the user is allowed to analyze the performance of each candidate deletion mutant according to these criteria and choose which gene deletion strategies will be the best fit for the experimental problem. The consensus ranking is obtained through the application of the rank product test [17, 18], which was previously successfully used for the meta-analysis of transcriptomes [19].

In detail, let *R*_*ij*_, with *i*=1,…,*N* and *j*=1,…,*C* be the rank of deletion *i* under the criteria *j*, *i.e.*, one sorts all the possible strategies/mutants according to that specific metric, from the best (*R*_*bestj*_=1) to the worst (*R*_*worstj*_=*N*). The rank product (RP) is defined as ${RP}_{i}=\prod _{j=1}^{C} R_{ij}$. The distribution of *R* *P*_*i*_ under the null hypothesis *H*_0_ of random ranks, which means that in each criteria the sorting is arbitrary, can be approximated with a Gamma distribution [17], determined exactly [18] and also approximated using the geometric means of upper and lower bounds [20]. This allows to calculate, for each deletion strategy, the *p*-value for *H*_0_ and identify which ones are statistically significant. Given the high number of hypotheses under study, a multiple testing correction must also be performed. To control the False Discovery Rate (FDR), we have used the *q*-value [21].

After this, all the possible deletions were sorted according to their *q*-values, in order to obtain a ranked list of putative best strategies to be further analyzed.

## Proposed pipeline

**Fig. 1:**
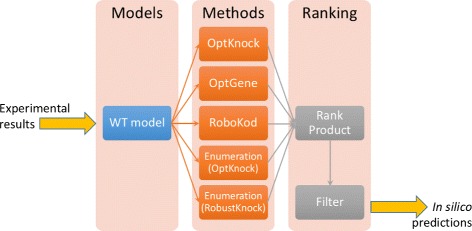
Data-flow diagram of the proposed pipeline

Below we present the developed pipeline, which is graphically depicted in Fig. 1. Further information about the procedure can be found in the Additional files 1 and 2. In vivo experimental measurements are considered as input, and are plugged in as model constraints. In order to account for the expected deviations in genetically engineered organisms, a 20% flexibility was allowed for both lower and upper bounds. The optimization methods are run after a pre-processing step, then the results are merged and ranked in order to generate a set of hypotheses, for further analysis and experimental testing.

### Pre-processing

Prior to applying the methods, a pre-processing step is executed in order to select a set of candidate reactions that may be deleted from the network. Then the methods are only run on the candidate reactions, which may significantly reduce the search-space and enable faster computation. After careful considerations and preliminary testing, the reactions falling into the following criteria are excluded from the candidate list thereof:

- **Essential reactions** were determined using flux balance analysis (FBA), maximizing the biomass on each possible mutant with single reaction deletion compared to the wild type (WT) model. If by deleting a reaction the problem gets infeasible (i.e. there is no solution) or if the maximal biomass is smaller than a threshold, the reaction is considered to be essential and cannot be deleted as a metabolic engineering strategy.
- **Blocked reactions** are carrying zero flux under any condition and are artifacts from the model reconstruction. This means that when maximizing or minimizing that particular flux, the value obtained is always zero as determined using flux variability analysis (FVA).
- **Synthetic and export reactions** are explicitly noted in the network and do not have a gene-reaction association. An example of this type of reaction is the import of substrate.

### Knockout prediction procedure

Three well established methods – OptKnock, OptGene and RobOKoD – are integrated into the pipeline. These methods are non-exhaustive, e.g. they return only one or a restricted set of solutions. The fourth method that is considered is based on an exhaustive enumeration (screening) of deletions, where a Flux Variability Analysis (FVA) is evaluated on all the possible gene deletions in order to calculate the maximal and minimal target compound productions given that the growth rate is optimal. Notice that enumeration of all deletions is only feasible if their maximum number considered is small. Obviously, this number depends on the model size and overall experimental constraints.

The first three methods present the advantage of being already extensively tested and experimentally validated [9, 14, 16]. The screening method allows the user to enumerate all the mutants and order them according to an optimistic and pessimistic prediction. The clear advantage is that the result is an exhaustive list of possible deletions, and different selection criteria can be applied to choose the best deletion strategy. For example, it might be more useful to select a strategy that has minimal guaranteed target production above zero, even if the maximal production is not the highest.

After the application of all these methods, the resulting lists are joined. The complete list of mutants is refined by filtering out those with a significant biomass loss. The threshold for growth rate was set to 0.1 h ^−1^. Also, results with zero maximal target production were not considered. This final list then undergoes a ranking step, where four measures are calculated for each mutant:

- **1. Maximal growth rate:** Computed with FBA.
- **2. Minimal target production:** The target compound is minimized given that the growth rate is maximal; computed with FVA.
- **3. Maximal target production:** The target compound is maximized given that the growth rate is maximal; computed with FVA.
- **4. Adaptability:** This measure indicates the distance between the mutant and the wild-type flux distributions based on the FVA of all fluxes in the network.

The first three measures are well established in the literature, see e.g. [22]. The rationale behind adaptability is that some gene deletion mutants can be predicted to have an increased production, but the flux distribution of the mutant is too different from the one of the wild type. The larger the difference, the more unlikely it is that the mutant will present that flux distribution in vivo, since it would imply a major metabolic readjustment that can be prevented by regulatory and enzymatic constraints. A similar biological consideration has motivated the development of the MOMA [13] and Regulatory On-Off Minimization (ROOM) [23] frameworks.

From the final list of mutants, the best candidates are retrieved, based on the four ranking criteria described above. The final list is sorted using the rank product method [17, 18], which is a biologically-motivated simple non-parametric statistical method test for combining ranked lists in various applications. Originally, it was introduced for the detection of differentially expressed genes in replicated microarray experiments [24], but has various application domains, including proteomics, metabolomics, statistical meta-analysis, and more generally, in feature selection. Since the growth and flux rates are sometimes the same for groups of deletions, the ranking is, in these cases, arbitrary inside these subsets. In order to identify potential biases when assigning the ranks in these cases, we reshuffled them and recalculated the permutation-based *q*-values. There were no significant changes on the final sorting list and values, and as such, we kept this strategy. There is some literature on partially ranked data and on Mallows models of permutation [25, 26] that can support the exact calculation of the *p*-values for lists with these characteristics. However, the development and application of such is out of the scope of this work and will be tested and analyzed in a future implementation of OptPipe.

To prevent a biased ranking stemming from parameters that have the same value for all the deletion mutants, a criterion was inserted enabling only parameters with a standard deviation above a given tolerance to be considered by the rank product method. In this case-study, no knockout strategy results in minimal target compound production higher than zero so this rate was disregarded.

## Implementation

The pipeline was implemented in MATLAB, based on the COBRA toolbox [4, 5] and with the external use of OptFlux [15]. The full detailed description and documentation of OptPipe can be found together with the software at https://github.com/AndrasHartmann/OptPipe along with all the source code.

The OptPipe software consists of three basic directories: (I.) common_functions contains the functions that are shared between the methods; (II.) methods contains the implementation of the main methods of the pipeline based on the COBRA toolbox, which is added to the (III.) external folder together with other libraries and toolboxes that are being used by the pipeline code, such as RobOKoD [16], RobustKnock [27] and xlwrite, that is a library for xls reading and writing. In addition, the (IV.) examples folder contains the working example of *Corynebacterium glutamicum* as described in the Results and discussion section. The OptGene methodology is not integrated into OptPipe, but runs externally in the OptFlux platform.

## Results and discussion

In order to test and validate the proposed method and software, OptPipe was applied to the optimization of naringenin production in *C. glutamicum*, a bacterium that is routinely used in the industrial production of amino acids [28]. Naringenin is a phenolic compound belonging to the flavonoids family that is naturally produced by some plants but is not endogenous in the *C. glutamicum* metabolism. This compound has interesting properties for health applications, such as being an antioxidant and chemoprotective [29, 30], and it also serves as a precursor for other flavonoids. Naringenin production from *p*-coumarate requires the expression of three heterologous genes, which code for 4-coumarate: CoA ligase (4CL), chalcone synthase (CHS) and chalcone isomerase (CHI). 4CL activates *p*-coumarate to its CoA-thioester *p*-coumaroyl-CoA. CHS then converts *p*-coumaroyl-CoA and three molecules of malonyl-CoA to naringenin chalcone, which is isomerized to naringenin by CHI. In *C. glutamicum*, the plasmid pMKEx2_*chsPh*_*chiPh* harbors the genes coding for CHS and CHI, while a 4CL-encoding gene is integrated into the genome of the tested *C. glutamicum* strain [31].

Malonyl-CoA is an endogenous metabolite derived from acetyl-CoA in *C. glutamicum*, and the maintenance of low levels of malonyl-CoA in this organism was identified as a major bottleneck during naringenin production. In order to test our method, the pipeline has been applied on the genome-scale metabolic model of *C. glutamicum* to find double deletion strategies that allow the strain to supply more malonyl-CoA for the production of naringenin.

### Model

The model *i*EZ475 for the strain ATCC 13032 of *C. glutamicum* was used, comprising 475 reactions and 408 metabolites of which 68 are extracellular and 340 are intracellular [32]. This model results from a process of extension and manual curation of the previously published *i*KK446 network [33]. The ability of the model to predict major physiological properties and the growth rate of different oxidative mutants has also been verified [32].

The SBML file for *C. glutamicum* *i*EZ475 and other connected resources such as the reaction and metabolite lists (including flux directions) as well as metabolic network map are available for download at http://www.13cflux.net.

### Predictions

After retrieving the individual rankings corresponding to the described criteria, we have obtained a list of 14 possible double deletion strategies that lead to the maximization of naringenin production (Table 1). All the proposed mutants have zero minimal target production, since the synthesis of naringenin is decoupled from growth. For this reason, minimal target production was not meaningful here, and was not used as a ranking criterion for the rank product methodology. The *ΔackAΔmetY* has the lowest *q*-value, with a predicted growth rate of 0.32 h ^−1^ and a maximal naringenin production of 0.5 mmol/gDCW/h, see gene annotations in the Additional files 1 and 2. Two other double deletion mutants (*ΔptaΔilvA* and *ΔptaΔmetY*) were found with a *q*-value of 0.1197. The *pta* and *ackA* reactions are associated in the model with a phosphate acetyltransferase and an acetate kinase, respectively, responsible for the diversion of acetyl-CoA towards acetate production. Taking into account the limiting character of the malonyl-CoA flux in naringenin optimization, it is unsurprising that the knockout of this pathway is expected to increase naringenin production. The remaining 11 double mutants had the same *q*-values, which in some of the cases is due to their equal characteristics and in other cases might reflect a trade-off between higher growth rates and lower distances between the mutant flux distribution and the wild-type. The results obtained with OptGene and RobustKnock were excluded from this list since they did not comply with the specified growth threshold (data not shown). See detailed results and gene annotation in the Additional files 1 and 2.

**Table 1:** Hypothesis deletions for enhancing naringenin production in *C. glutamicum* and corresponding sorting criteria

Strain	Growth rate	min Nar	max Nar	Distance to WT	*p*-value	*q*-value	Method
	(*h* ^−1^)	(mmol/gDCW/h)	(mmol/gDCW/h)	(mmol/gDCW/h)			
wild type	0.50	0	0				
*Δ* *ackA* *Δ* *metY*	0.32	0	0.50	400	0.0742	0.0779	OptKnock ^a^
*Δ* *pta* *Δ* *ilvA_ile*	0.20	0	0.50	404	0.2432	0.1197	
*Δ* *pta* *Δ* *metY*	0.32	0	0.50	400	0.2342	0.1197	
*Δ* *ackA* *Δ* *ilvA_ile*	0.20	0	0.50	404	0.4139	0.1285	
*Δ* *ackA* *Δ* *metY*	0.32	0	0.50	400	0.3656	0.1285	
*Δ* *sdhCAB* *Δ* *ddh*	0.21	0	0.50	545	0.5443	0.1285	OptKnock
*Δ* *mdh* *Δ* *pyc*	0.23	0	0.49	39	0.5377	0.1285	screening
*Δ* *pyc* *Δ* *zwf*	0.13	0	0.50	565	0.8300	0.1285	
*Δ* *pyc* *Δ* *opcA*	0.13	0	0.50	565	0.8580	0.1285	
*Δ* *pyc* *Δ* *gnd*	0.13	0	0.50	565	0.8784	0.1285	
*Δ* *pyc* *Δ* *rpe*	0.16	0	0.50	441	0.7986	0.1285	
*Δ* *pyc* *Δ* *tkt_1*	0.15	0	0.50	532	0.8553	0.1285	
*Δ* *pyc* *Δ* *tal*	0.15	0	0.50	532	0.8940	0.1285	
*Δ* *pyc* *Δ* *tkt_2*	0.17	0	0.50	413	0.8340	0.1285	
*Δ* *pyc* *Δ* *pmmB*	0.19	0	0.17	47	0.7249	0.1285	

^a^OptKnock result corresponding to the (single) obtained solution

**Fig. 2:**
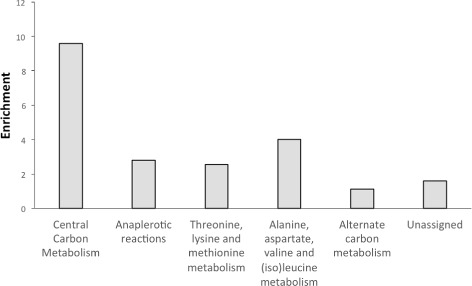
Enrichment of the KOs proposed to increase naringenin production. Each reaction of the model was classified in a category of metabolic pathway, the frequency each category in the KO dataset was computed and normalized for the frequency of each category in the model

The proposed reaction deletions were categorized into main metabolic subsystems. The enrichment of the overall knockout dataset was computed to understand which sections of the bacterial metabolism were mostly identified as knockout targets when optimizing naringenin production (Fig. 2). Out of the 14 subsystems present in the model, the reactions selected were concentrated in six: 1) central carbon metabolism; 2) anaplerotic reactions; 3) threonine/lysine/methionine metabolism; 4) alternate carbon metabolism; 5) alanine/aspartate/(iso)leucine metabolism; and 6) unassigned reactions. The most enriched category was the central carbon metabolism, comprising succinate dehydrogenase (*sdhCAB*) and various reactions from the pentose phosphate pathway (*zwf*, *opcA*, *rpe*, *tkt_.1*, *tal*, *tkt_.2* and *gnd*). These pathways can be viewed as major carbon drains, diverting glucose and acetyl-CoA towards the production of reducing equivalents and amino acid precursors.

Enzymes catalyzing anaplerotic reactions, such as pyruvate carboxylase (*pyc*), with a role in gluconeogenesis, were also identified in the knockout set.

### Validation

When considering the cultivation and production conditions necessary for microbial polyphenol synthesis with *C. glutamicum*, several deletions could be omitted from the set. Among these were e.g. all combinations comprising deletions of either *pta* or *ackA*, which would only prevent the loss of acetyl-CoA as an important malonyl-CoA precursor if the cultivation conditions promote acetate formation. In addition, combinations suggesting the deletion of *pyc* were also not considered as the predicted growth rate was simply too low to comply with requirements of microbial production in the aspired industrial setting.

**Fig. 3:**
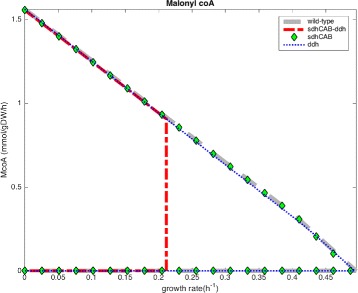
Production envelope for *C. glutamicum* mutants with enhanced malonyl-CoA production. Obtained with the internal Cobra Toolbox function

It was found that the *ΔsdhCABΔddh* (succinate dehydrogenase and diaminopimelate dehydrogenase) deletions can also have biological significance, and may constitute promising targets for testing in vivo [34]. The envelope of Fig. 3 shows the most promising predicted deletion strategies to enhance the malonyl-CoA supply for naringenin production. Batch fermentation validation experiments were set up, including the single *ΔsdhCAB* and *Δddh* mutants, the double *ΔsdhCABΔddh* mutant and the reference strain. Naringenin titer and biomass were measured.

**Fig. 4:**
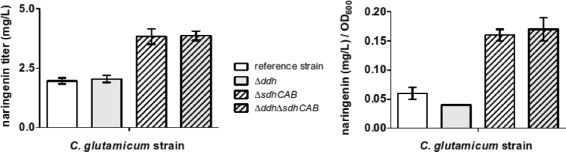
Naringenin production of the *C. glutamicum* strains. The obtained titers of naringenin and the biomass-normalized yield are shown for the constructed strains harboring the plasmid pMKEx2_chs _Ph__chs _Ph_. The final biomass (OD_600_) values obtained after 48 hours were 35.6±1.3 (reference strain), 45.2±1.1 (*Δddh* strain), 24.1±0.3 (*ΔsdhCAB* strain) and 22.3±2.1 (*ΔddhΔsdhCAB* strain). Data represent average values and standard deviation obtained from three biological replicates

The validation experimental results summarized in Table 2 and Fig. 4 show that deletion of the *ddh* gene did not increase naringenin titer, and even slightly decreased naringenin levels per OD unit. On the other hand, the *ΔsdhCAB* mutant showed significant improvement of naringenin production, and a two-fold increase in normalized yield, while the loss in biomass was less than 10% lower than what was predicted. Interestingly, the double deletion strain *ΔsdhCABΔddh* behaves similar to the single deletion strain (*ΔsdhCAB*) with respect to all of the measured features.

**Table 2:** Biomass and optical density of the constructed gene deletion strains

Strain	Growth rate	Final biomass
(without plasmid)	*μ*_max_ (*h* ^−1^)	(OD_600_)
*C. glutamicum* DelAro^4^- 4*c* *l*_*Pc*_	0.34±0.01	55.2±1.7
*C. glutamicum* DelAro^4^- 4*c* *l*_*Pc*_ *Δ* *d* *d* *h*	0.35±0.01	54.3±0.4
*C. glutamicum* DelAro^4^- 4*c* *l*_*Pc*_ *Δ* *s* *d* *h* *C* *A* *B*	0.27±0.02	28.1±0.7
*C. glutamicum* DelAro^4^- 4*c* *l*_*Pc*_ *Δ* *d* *d* *h* *Δ* *s* *d* *h* *C* *A* *B*	0.27±0.01	29.2±0.1

The maximal growth rate and the final biomass were analyzed for the constructed gene deletion strains not harboring the plasmid pMKEx2_chs _Ph__chs _Ph_. The cultivation was performed in absence of plasmid and inducer of plasmid-borne gene expression to avoid any overlapping effects on growth behavior either resulting from the deleted genes or from differences in obtained naringenin titers/ expression of heterologous genes. Data represent average values and standard deviation obtained from three biological replicates

In summary, the in vivo experiments confirmed the beneficial effects of the *ΔsdhCAB* deletion, while the *Δddh* deletion did not increase the naringenin production any further. The difference between the predicted and the validated results as concerns naringenin and growth could be due to the incompleteness of the wild-type strain model. Indeed, this may lead to a simulation of the behaviour of the strain DelAro4 that is not fully accurate. A modification of the model and of the respective constraints might be beneficial, however this is not trivial and is thus out of the scope of the current study.

## Conclusion

Different optimization methods in metabolic engineering often lead to conflicting results that can become difficult to interpret. We propose OptPipe - a Pipeline for Optimizing Metabolic Engineering Targets, based on a consensus approach. The method is based on combining several optimization solutions obtained from distinct algorithms and ranking them according to several objectives, such as biomass and target production. In addition, an adaptability measure was introduced to account for the likelihood of a predicted flux distribution for a mutant to occur in vivo with the kinetic and regulatory constraints found within the cell. OptPipe was applied to a case-study of maximizing malonyl-CoA production using the genome-scale metabolic model of *C. glutamicum*, where one predicted knockout of succinate dehydrogenase (*ΔsdhCAB*) did result in an increased production in vivo. This however only represents an isolated case, and further experimental validation of the methodology is required in order to reach conclusion about the efficiency of the rank product method to distinguish between promising and less promising knockout strategies.

The utility of the proposed pipeline is associated with allowing the user to simultaneously obtain a list of possible strategies that are derived from methods based on different rationales. In this setting, the pessimistic and optimistic approaches of OptKnock and RobustKnock are paradigmatic. In this way, the user may browse through the proposed knockouts and evaluate their feasibility based on their performance in the rank product test. Furthermore, OptPipe is a general framework that can be applied to any organism and target product, and significantly supports metabolic engineering tasks.

## Availability and requirements

**Project name:** OptPipe

**Project home page:** https://github.com/AndrasHartmann/OptPipe

**Operating system(s):** Platform independent

**Programming language:** MATLAB (Tested on 2015a and 2016b)

**Other requirements:**

- MATLAB parallel toolbox
- COBRA toolbox from the openCOBRA project (release 2.0),
- SBML toolbox,
- Gurobi optimization software installed for MATLAB
- OptFlux (recommended)

**License:** GPL v3

**Any restrictions to use by non-academics:** none

**Install:** See detailed description on the project home page

## Additional files

Additional file 1 OptPipe - Supplementary Methods. Optimization algorithms and pipeline description. (PDF 189 kb)

Additional file 2 OptPipe - Supplementary Results. Additional Tables. (XLSX 221 kb)
