# Supplementary material for: OptPipe - a pipeline for optimizing metabolic engineering targets
Source: BMC Syst Biol. 2017 Dec 21;11:143. doi: 10.1186/s12918-017-0515-0 (PMC5740890; doi:10.1186/s12918-017-0515-0)
Supplement: Supplementary file 1 — OptPipe - Supplementary Methods. Optimization algorithms and pipeline description. (PDF 189 kb) [file 12918_2017_515_MOESM1_ESM.pdf]

OptPipe - a Pipeline for Optimizing Metabolic Engineering Targets

Supplementary Material

A. Hartmann, A. Vila Santa, N. Kallscheuer, M. Vogt, A. Julien-Laferrière, M.-F. Sagot, J. Marienhagen and S. Vinga

September 2017

1 Optimization methods

The different optimization methods that have been considered for knockout prediction on the genome-scale models are the following: OptKnock, RobustKnock, MOMA, OptGene and RobOKoD. The Table below contains the references and availability of the different methods.

Method	Reference	Availability
OptKnock	[1]	COBRA Toolbox [2] (open-source)
RobustKnock	[3]	Author's webpage
MOMA	[4]	COBRA Toolbox [2] (open-source)
OptGene	[5, 6]	OptFlux [7] (open-source)
RobOKoD	[8]	Supplementary material
Enumeration methods	This study	OptPipe

1.1 OptKnock

OptKnock gives an optimistic prediction to the target production based on the premise that the organism maximizes the biomass [1]. The problem is formulated as a bi-level

mixed integer linear problem (MILP)

$$
\begin{aligned}
&\underset{y_j}{\text{maximize}} \quad v_{prod} \\
&\text{subject to} \\
&\qquad \begin{bmatrix}
\underset{v_j}{\text{maximize}} & v_{biomass} & \\
\text{subject to} & & \\
& \sum_{j \in J} S_{ij} v_j = 0 & \forall i \in I \\
& LB_j(1-y_j) \leq v_j \leq UB(1-y_j), & \forall j \in J
\end{bmatrix} \\
&\qquad \sum_{j \in J} y_j \leq \kappa \\
&\qquad y_j \in \{0,1\}, \; v_j \in \mathbb{R}, \quad \forall j \in J
\end{aligned} \tag{1}
$$

Where v_{prod} is the target compound to produce, $v_{biomass}$ is the reaction corresponding to the biomass, S is the stoichiometric matrix, LB and UB the lower and upper bounds, respectively. The variables of the inner problem are the fluxes v, and the outer problem operates on the binary variables, y, representing the deletions. Here the value $y_j = 1$ means that the jth reaction is deleted and $y_j = 0$ has the meaning that it is not. The maximum number of deletions is κ. It was shown in [1, 9] that the bi-level optimization problem can be translated into a single-level MILP. State-of-the art mixed integer solvers like gurobi and CPLEX can find a globally optimal solutions of such problems.

1.2 RobustKnock

RobustKnock is formulated as a three-level mixed-integer optimization problem [3] which, similarly to the OptKnock, can be transformed to a single-level mixed integer problem. Here the outer two levels comprise a max-min problem, which is a common formulation for loss minimization, optimizing for the worst-case scenario.

$$
\begin{aligned}
&\underset{y_j}{\text{maximize}} \quad \underset{v_j}{\text{minimize}} \quad v_{prod} \\
&\text{subject to} \\
&\qquad \begin{bmatrix}
\underset{v_j}{\text{maximize}} & v_{biomass} & \\
\text{subject to} & & \\
& \sum_{j \in J} S_{ij} v_j = 0 & \forall i \in I \\
& LB_j(1-y_j) \leq v_j \leq UB(1-y_j), & \forall j \in J
\end{bmatrix} \\
&\qquad \sum_{j \in J} y_j \leq \kappa \\
&\qquad y_j \in \{0,1\}, \; v_j \in \mathbb{R}, \quad \forall j \in J
\end{aligned} \tag{2}
$$

1.3 OptKnock vs. RobustKnock

In a sense, OptKnock represents an optimistic prediction because when fixing the binary variables, it formulates the question: "What is the maximal compound production given that the organism optimizes for biomass?" By the same token, RobustKnock serves as a pessimistic prediction for the biomass-coupled target production, *i.e.* it maximizes the the minimal target compound given that the biomass is maximized. See Figure 1 for an illustrative example on the two different approaches.

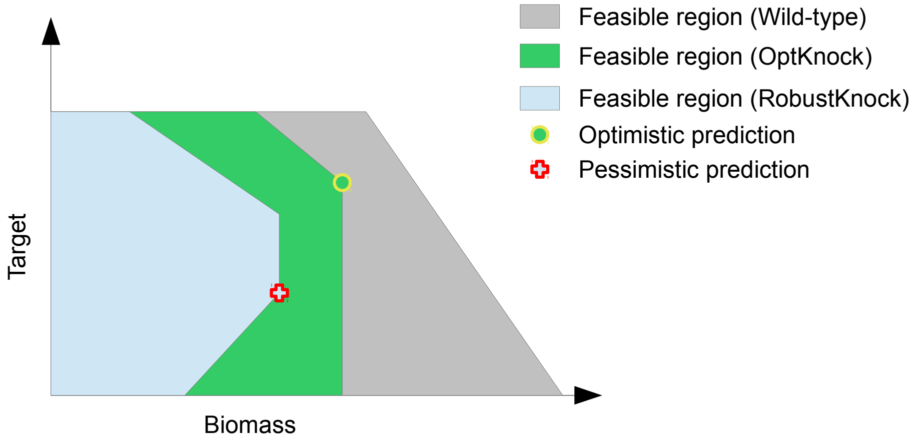

Figure 1: Examples of the feasible regions of OptKnock and RobustKnock compared to the wild-type. Also the optimistic (OptKnock) and pessimistic (RobustKnock) predictions are shown.

1.4 MOMA

Minimization Of Metabolic Adjustment (MOMA) relies on the assumption that the fluxes of the genetically engineered organism undergo a minimal redistribution when compared to the wild type organism as a reference flux distribution [4]. This is expressed in the optimization problem minimizing the distance of the flux distribution of the mutant to the reference.

$$\begin{aligned} \underset{v_j}{\text{minimize}} \quad & \ell(v - v_{ref}) \\ \text{subject to} \quad & \\ & \sum_{j \in J} S_{ij} v_j = 0 \qquad\qquad \forall i \in I \\ & LB_j(1 - y_j) \leq v_j \leq UB(1 - y_j), \quad \forall j \in J \end{aligned} \tag{3}$$

Where $\ell(\cdot)$v is a distance measure. Because of shorter running-time, in our applications the linear version of MOMA (lMOMA) was used, which involves the minimization of

the first norm between the reference organism and the modified one. The reference is represented by the parsimonious Flux Balance Analysis (pFBA), consisting of two steps. First a traditional Flux Balance Analysis (FBA) is done in order to calculate the wild type biomass denoted by f.

$$\begin{aligned} f = \underset{v}{\text{maximize}} \quad & v_{biomass} \\ \text{subject to} \quad & \\ & \sum_{j \in J} S_{ij} v_j = 0 \qquad \forall i \in I \\ & LB_j \leq v_j \leq UB, \quad \forall j \in J \end{aligned} \tag{4}$$

$$\begin{aligned} \underset{v_{ref}}{\text{minimize}} \quad & |v_{ref}| \\ \text{subject to} \quad & \\ & v_{biomass} = f \\ & \sum_{j \in J} S_{ij} v_{ref_j} = 0 \qquad \forall i \in I \\ & LB_j \leq v_{ref_j} \leq UB, \quad \forall j \in J \end{aligned} \tag{5}$$

1.5 OptGene

OptGene [5, 6] considers the application of Evolutionary Algorithms (EAs) and Simulated Annealing (SA). Unlike OptKnock and RobustKnock that guarantee global optimal solutions, EA and SA are meta-heuristic methods that are only capable of providing near-optimal solutions but within a reasonable computation time. Another benefit of OptGene is being quite flexible regarding the objective function that can be optimized (*e.g.* they are able to deal well with non linear functions). A popular choice is the Biomass-Product Coupled Yield (BPCY) [5], where is the flux of the substrate which serves as precursor for the target compound.

1.6 RobOKoD

RobOKoD identifies knockout, overexpression/dampening targets in an iterative three-step process [8]. First a Metabolite Consumption Test (MCT) is executed to identify metabolites within the optimal target production pathway that are also consumed to produce biomass, and to determine their promiscuity. The second step is a flux variability analysis profiling (FVAp), where the reactions are profiled to determine which reaction would make an ideal knockout / dampening / overexpression target. The third step is a selection of the most promising deletions. The strength of RobOKoD is that it is able to predict and rank knockouts, over- expressions, and dampening targets. While predicting an optimized set of gene modifications to implement, unlike other methods, RobOKoD also provides lists of candidate modifications, along with graphical flux variability profiles, allowing the user to manually validate the set of predictions.

2 Detailed pipeline

The step-by-step procedure applied in the pipeline is detailed below:

1. Introduction of experimental constraints for a wild-type *C. glutamicum* strain. These values comprise the consumption rates of glucose, acetate and oxygen and production rates of succinate and carbon dioxide, and were obtained from [10]. Additionally, for the particular case study of naringenin production in *C. glutamicum* it was considered that 5-coumarate is provided in the media, to mimic the experimental conditions.

2. Define the set of reactions that should not be considered for deletion ("Irrelevant reactions")
   a) Essential reactions: required for growth. This set was determined by evaluating every single reaction deletion and the corresponding growth rate; deletions which resulted in a growth rate below a given threshold (10^{-9}) were considered lethal.
   b) Transport and synthetic reactions: transport, sink and macromolecule formation reactions. This list was compiled based on a search by reaction name.
   c) Blocked reactions: cannot carry a non-null flux and are therefore, an artifact from the reconstruction process. This set was compiled through the use of the `findBlockedReaction`, built in the Cobra Toolbox.

3. Optimization methods (see formal descriptions above):
   a) OptKnock
   b) RobOKoD
   c) OptGene. The use of OptGene within the Cobra Toolbox required the commercial solver `tomlab`; to avoid this, the OptGene was run manually in the OptFlux workbench. To this end, the model was exported in the SBML format, imported in the workbench and the results were manually written in a `.xlsx` file to be then imported into the pipeline in Matlab environment. The parametrization of the method is listed below

Parameter	Value
Simulation Method	MOMA
Objective Function	BPCY, considering 5-coumarate as the substrate
Simulation algorithm	SPEA2
Number of solution evaluations	5000
Number of knockouts	3

To avoid OptFlux from considering "Irrelevant Reactions" to knockout, the gene association was removed in these reactions and the evolutionary algorithm was run on the gene deletion option, instead of the reaction deletion option.

d) Enumeration using the OptKnock objective function

e) Enumeration using the RobustKnock objective function

In both enumeration approaches the mutants with a growth rate below a tolerance of 10^{-9} were disregarded.

4. Join the knockout lists generated by the 5 methods

5. Compute the 4 parameters that will be used for the ranking procedure for each deletion mutant combination

   a) Maximal growth rate

   b) Minimal target production

   c) Maximal target production

   d) Adaptability, *i.e.* the (sum of absolute differences) distance between the flux distribution of the mutant and the wild-type flux variability. For each reaction in the network, the distance between the flux in the deletion mutant distribution and the minimal/maximal flux of the wild-type distribution is calculated. Differences less than 10% of the minimal wild-type flux were considered insignificant and excluded from the final sum of the differences of all the fluxes, yielding the adaptability parameter.

6. Rank Product (RP) application in 10 iterations to account for randomness in rank attribution between deletion mutants that are tied for a given parameter. Parameters that have a standard deviation below 10^{-5} are not considered for the method.

7. Filtering of proposed deletion strategies that have a growth rate below 0.1 and a minimal target production of zero.

References

[1] Burgard, A.P., Pharkya, P., Maranas, C.D.: Optknock: a bilevel programming framework for identifying gene knockout strategies for microbial strain optimization. Biotechnology and bioengineering **84**(6), 647–57 (2003). doi:10.1002/bit.10803

[2] Schellenberger, J., Que, R., Fleming, R.M.T., Thiele, I., Orth, J.D., Feist, A.M., Zielinski, D.C., Bordbar, A., Lewis, N.E., Rahmanian, S., Kang, J., Hyduke, D.R., Palsson, B.Ø.: Quantitative prediction of cellular metabolism with constraint-based models: the COBRA Toolbox v2.0. Nature protocols **6**(3), 1290–1307 (2011). doi:10.1038/nprot.2011.308

[3] Tepper, N., Shlomi, T.: Predicting metabolic engineering knockout strategies for chemical production: Accounting for competing pathways. Bioinformatics **26**(4), 536–543 (2009). doi:10.1093/bioinformatics/btp704

[4] Segrè, D., Vitkup, D., Church, G.M.: Analysis of optimality in natural and perturbed metabolic networks. Proceedings of the National Academy of Sciences of the United States of America **99**(23), 15112–7 (2002). doi:10.1073/pnas.232349399

[5] Patil, K.R., Rocha, I., Förster, J., Nielsen, J.: Evolutionary programming as a platform for in silico metabolic engineering. BMC Bioinformatics **6**, 308 (2005). doi:10.1186/1471-2105-6-308

[6] Rocha, M., Maia, P., Mendes, R., Pinto, J.P., Ferreira, E.C., Nielsen, J., Patil, K.R., Rocha, I.: Natural computation meta-heuristics for the in silico optimization of microbial strains. BMC Bioinformatics **9**, 499 (2008). doi:10.1186/1471-2105-9-499

[7] Rocha, I., Maia, P., Evangelista, P., Vilaça, P., Soares, S., Pinto, J.P., Nielsen, J., Patil, K.R., Ferreira, E.C., Rocha, M.: OptFlux: an open-source software platform for in silico metabolic engineering. BMC Systems Biology **4**, 45 (2010). doi:10.1186/1752-0509-4-45

[8] Stanford, N.J., Millard, P., Swainston, N.: RobOKoD: microbial strain design for (over)production of target compounds. Frontiers in Cell and Developmental Biology **3**(March), 1–12 (2015). doi:10.3389/fcell.2015.00017

[9] Chowdhury, A., Zomorrodi, A.R., Maranas, C.D.: Bilevel optimization techniques in computational strain design. Computers and Chemical Engineering **72**, 363–372 (2014). doi:10.1016/j.compchemeng.2014.06.007

[10] Zelle, E., Nöh, K., Wiechert, W.: Interrogating a Genome-scale Metabolic Network Model. In: Burkovski, A. (ed.) Growth and Production Capabilities of Corynebacterium Glutamicum, 1st edn., pp. 39–56. Caister Academic Press, ??? (2015). Chap. 4
